# Cardiac arrest in seronegative idiopathic inflammatory myopathy: a case report

**DOI:** 10.1093/ehjcr/ytad589

**Published:** 2023-11-24

**Authors:** Varun Srivatsav, Ambreen Khan, Stephan Wardell

**Affiliations:** Division of Cardiology, Department of Medicine, Queen’s University, Armstrong 3, Kingston General Hospital, 76 Stuart St, Kingston, ON, K7L 2V7, Canada; Division of Rheumatology, Department of Medicine, University of Saskatchewan, College of Medicine, 107 Wiggins Rd, Saskatoon, SK, S7N 5E5, Canada; Division of Cardiology, Department of Medicine, University of Saskatchewan, College of Medicine, 107 Wiggins Rd, Saskatoon, SK, S7N 5E5, Canada

**Keywords:** Ventricular fibrillation, Cardiomyopathy, Cardiac risk, Case report, Myocarditis, Autoimmune inflammatory myopathies

## Abstract

**Background:**

Idiopathic inflammatory myopathies (IIMs) are autoimmune diseases that are characterized by muscle injury. These disorders can cause cardiomyopathy and heart failure, myocarditis, and arrhythmias. However, only a few cases of cardiac arrest as a result of IIMs have been previously reported.

**Case summary:**

A 46-year-old male presented with an out-of-hospital ventricular fibrillation cardiac arrest. A diagnosis of IIM had been made through a muscle biopsy performed 2 years before presentation. The patient had a positive anti-nuclear antibody but negative myositis-specific antibodies. His initial symptoms of IIM were mild and consisted of myalgias. His only cardiac symptoms were minor palpitations that occurred 3 years prior to the cardiac arrest, with a negative Holter monitor test result at that time. His cardiac catheterization was normal. He was suspected to have myocarditis, and a rheumatologist was consulted, following which the patient was initiated on intravenous immunoglobulin (IVIG). Cardiac magnetic resonance imaging demonstrated evidence of chronic myocarditis and an ejection fraction of 44%. He was initiated on goal-directed medical therapy for heart failure. A VVI implantable cardioverter defibrillator was implanted for secondary prevention. He was discharged and prescribed additional immunosuppression including further IVIG infusions, prednisone taper and rituximab infusions.

**Discussion:**

Our case demonstrates that cardiac arrest in IIM is not only plausible, but can be the first major cardiac manifestation of the disease. When a diagnosis of IIM is made, patients require a thorough assessment of cardiac symptomatology and a low threshold for additional cardiac investigations.

**Learning points**

- To understand the nature of myocardial involvement in idiopathic inflammatory myopathies.
- To raise awareness around the importance of screening patients with inflammatory myopathies for cardiac involvement.

## Primary specialities involved other than cardiology

Rheumatology, internal medicine, radiology.

## Introduction

Idiopathic inflammatory myopathies (IIMs) are characterized by muscle injury caused by immunological phenomena.^1^ Specific IIMs include dermatomyositis, polymyositis, inclusion body myositis, and necrotizing autoimmune myopathy, with each one having distinct clinical and histological characteristics.^2^ These rare disorders cause muscle weakness, elevated muscle enzymes, and inflammation on muscle biopsy.^1,3^ IIMs are often associated with specific autoantibodies, which vary depending on the disease, although they are not required for the diagnosis. IIMs can also cause cardiac dysfunction consisting of cardiomyopathy and heart failure, myocarditis, conduction system disease, and tachyarrhythmias,^1,4,5^ but only a few cases presenting as cardiac arrest have been reported to date.^3,6,7^ Here in, we report a case of myocarditis secondary to an undifferentiated seronegative IIM presenting as a ventricular fibrillation (VF) cardiac arrest.

## Summary figure

**Table:** 

Two years prior to the arrest	Diagnosed with idiopathic inflammatory myopathy (IIM) through muscle biopsy; disease well controlled on azathioprine with no significant cardiac symptoms
Day 0	Admitted for out-of-hospital ventricular fibrillation cardiac arrest. Cardiac catheterization demonstrated no significant coronary artery disease. Rheumatology consultation was made for presumed myocarditis secondary to IIM
Day 1	Intravenous immunoglobulin induction therapy initiated for presumed myocarditis
Day 3	Patient had a successful neurological recovery and was extubated. Initiated on goal-directed medical therapy for heart failure with mid-range ejection fraction
Day 7	Cardiac magnetic resonance imaging (MRI) confirmed chronic myocarditis
Day 9	Secondary prevention VVI implantable cardioverter defibrillator inserted
Day 10	Patient discharged with close cardiology and rheumatology follow-up and plan for initiation of further immunosuppression
One-year post-arrest	Patient is currently asymptomatic and creatine kinase level has normalized. Continued immunosuppression with rituximab and azathioprine with repeat cardiac MRI pending

## Case summary

**Figure 1:**
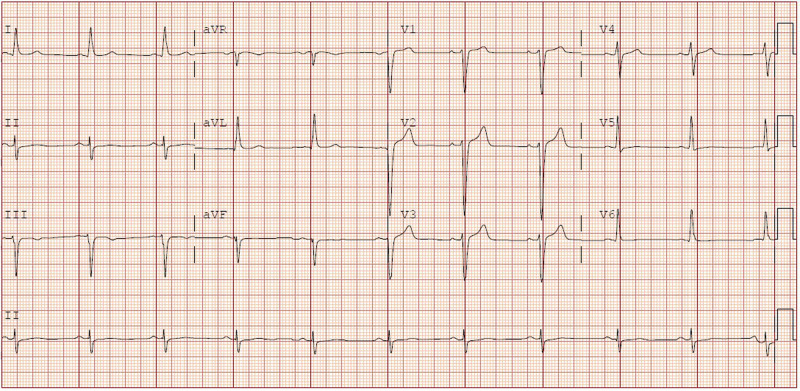
Baseline electrocardiogram 1 year prior to the cardiac arrest. A sinus rhythm with left axis deviation and late R wave transition. QRS duration 117 ms, PR interval 142 ms, and corrected QT interval 414 ms.

A 46-year-old Caucasian male with a history of undifferentiated seronegative IIM suffered an out-of-hospital cardiac arrest. He was previously well and was asymptomatic from a cardiac perspective. While curling, he lost consciousness and was found to be pulseless; immediate bystander CPR was initiated. Upon arrival of emergency services, he received defibrillation twice for VF with a subsequent return of spontaneous circulation, six minutes of downtime. He had a past history of IIM diagnosed two years prior to the arrest from a quadriceps muscle biopsy. Initial symptoms at the time were fatigue and myalgias, but no muscle weakness or rashes were noted. He had no significant previous cardiac symptoms, except for infrequent palpitations, for which a 24 h Holter monitor was done three years prior to the arrest; the test result was unremarkable. After the diagnosis of IIM, he did undergo a baseline electrocardiogram (ECG) one year prior to the arrest, which did reveal a sinus rhythm with left axis deviation and late R wave transition in the precordial leads (*Figure 1*). Baseline echocardiogram performed one year prior to the arrest demonstrated an ejection fraction (EF) of 55–60% and a focal dyskinetic segment in the basal inferolateral wall. His creatine kinase (CK) levels ranged from 1100 to 2600 IU/L (normal 20–215 IU/L) for three years prior to the cardiac arrest. The result of an anti-nuclear antibody test performed three years prior to the arrest was positive with a titre of 1:1280 and a speckled pattern, but the results of his tests for myositis-specific antibodies were negative. He was on azathioprine 100 mg daily before presentation, and given minimal symptoms and improvement in his initial CK, the disease was thought to have been well controlled. Additional history included papillary thyroid carcinoma with complete curative thyroidectomy performed three years prior to the cardiac arrest, anxiety, and gastroesophageal reflux disease. The patient worked as a mortgage broker with no significant environmental risk factors. He was fully compliant with his medications. There was no history of previous viral infections or of COVID-19. Also, no family history of dilated cardiomyopathy or sudden cardiac death was reported.

**Figure 2:**
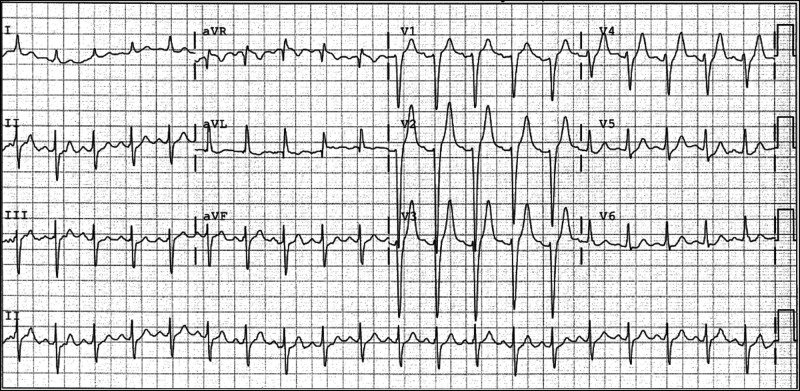
Initial electrocardiogram during cardiac arrest presentation. A sinus tachycardia with left axis deviation and late R wave transition. QRS duration 118 ms, PR interval 145 ms, and corrected QT interval 450 ms.

In the emergency department, measurements of vital signs revealed an oxygen saturation of 86% on room air, heart rate of 115 b.p.m., blood pressure of 164/97 mmHg, and temperature of 36.4°C. He was intubated. Examination revealed no extra heart sounds or murmurs. A neurological examination revealed a Glasgow coma scale of 3. The pupils were equal and reactive, and brainstem reflexes were present. Head, neck, respiratory, and abdominal examination results were unremarkable. ECG on presentation revealed a sinus tachycardia with left axis deviation and late R wave transition (*Figure 2*).

Laboratory investigations revealed a high-sensitivity troponin T level of 220 ng/L (normal range <5 ng/L), blood gas with pH 6.89 (normal range 7.35–7.45), pCO_2_ 66 mmHg (normal range 35–45 mmHg), a bicarbonate level of 13 mmol/L (normal range 23–29 mmol/L), and a lactate level of 19 mmol/L (normal range <2 mmol/L). The N-terminal propeptide B-type natriuretic peptide (NT-pro-BNP) level was 269 pg/mL (normal range <300 pg/mL for age <50). The C-reactive protein level was 2.5 mg/L (normal range <10 mg/L), the erythrocyte sedimentation rate was 17 mm/h (normal range 0–10 mm/h), and the CK level was 1233 U/L (normal 20–215 IU/L). For serology, the test results of rheumatoid factor, anti-cyclic citrullinated peptide, anti-nuclear cytoplasmic antibody, complement 3 and 4, and cryoglobulin were negative. Immunoglobulins were within the normal range. Anti-nuclear antibody test result was positive with a mixed pattern containing a titre of 1:80 with a few discrete nuclear dots and a titre of 1:160 with a nuclear envelope. Anti-SSA, anti-SSB, anti-ribonucleoprotein, and anti-smith antibody test results were negative. The test results of COVID-19, cytomegalovirus, Epstein–Barr virus, and respiratory virus panel were also negative.

Chest X-ray was unremarkable. A coronary angiogram demonstrated no coronary artery disease. At that point, targeted temperature management was initiated with cooling to 33°C. A rheumatologist was consulted, and with a presumed diagnosis of myocarditis secondary to an undifferentiated IIM, intravenous immunoglobulin (IVIG) was initiated at 2 g/kg over 4 days.

**Figure 3:**
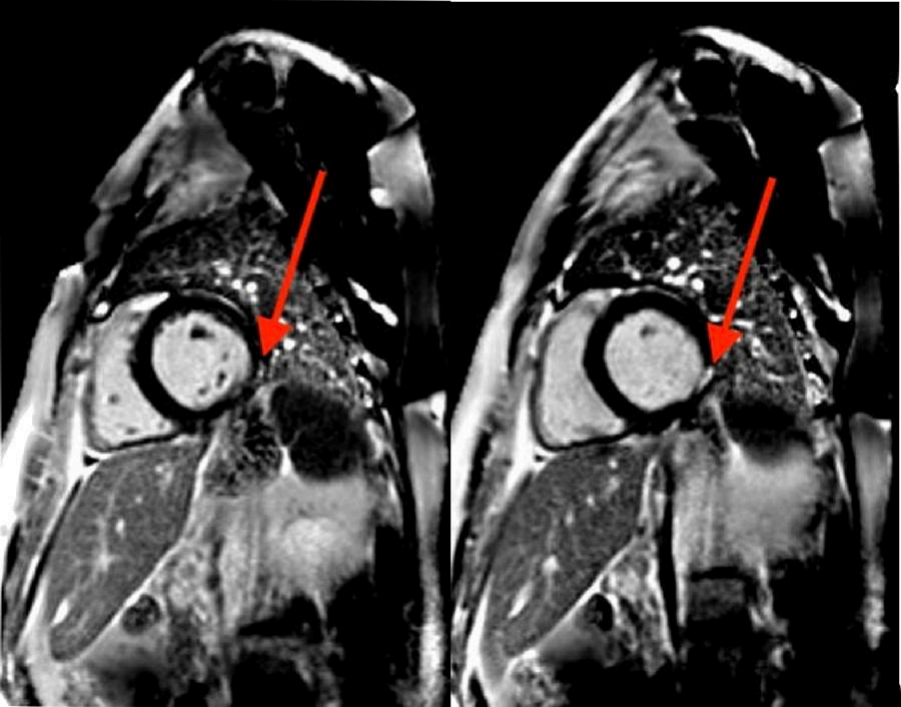
Cardiac magnetic resonance imaging. MRI short-axis views demonstrated late gadolinium enhancement of the basal inferolateral wall that was predominantly mid-myocardial. This finding can be seen in chronic myocarditis. T2 imaging result was negative.

A transthoracic echocardiogram revealed an EF of 45–50% with hypokinesis of the basal inferolateral wall (see Supplementary material online, *Video S1*). A cardiac MRI (cMRI) revealed late gadolinium enhancement (LGE) of the basal inferolateral wall of the left ventricle (*Figure 3*). The region of scarring was mid-myocardial and suggestive of chronic myocarditis. There were no other regions of scarring. There was no increased T2 signal to suggest oedema or active inflammation. The left ventricle (LV) was normal in size and the EF was 44% with mild global hypokinesis (see Supplementary material online, *Video S2*). Other cardiac structures, including the right ventricle, valves, and pericardium, were normal.

The patient made a successful neurological recovery and was extubated on the third day post-arrest. Electrophysiology was involved for the placement of a VVI implantable cardioverter defibrillator (ICD). The patient was initiated on carvedilol, ramipril, spironolactone, and dapagliflozin as part of goal-directed medical therapy for heart failure since the patient had a mid-range EF, to further improve left ventricular function. The patient was continued on IVIG 2 g/kg each month for 6 months. Upon discharge, for further immunosuppression, the patient was initiated on a prednisone taper at 50 mg daily for 2 weeks, and the dosage was decreased by 5 mg every 2 weeks.

In the initial follow-up, the patient was initiated on rituximab 1 g every 6 months to be continued for 2 years. The patient was also re-initiated on azathioprine 100 mg daily for the maintenance of immunosuppression. In continued follow-up 1 year after the cardiac arrest, the patient had become asymptomatic, had returned to work, and his CK level had normalized. His follow-up echocardiogram demonstrated a similar left ventricular function to previous with an EF of 45%, with hypokinesis of the basal inferolateral wall (see Supplementary material online, *Video S3*). The patient will be closely followed up by clinicians of the cardiology and rheumatology departments, and a repeat cMRI will be obtained for reassessment.

## Discussion

Limited data relating to the cardiac complications of IIM are available, but recent literature demonstrates that cardiac involvement is significantly more common than previously identified, often presenting with heart failure, cardiomyopathy, myocarditis, conduction abnormalities, and tachyarrhythmias.^3,6^ In the Euromyositis registry, published in 2018, it has been reported that 9% of 1715 IIM patients had cardiac involvement.^3,8^ Cardiac involvement is often subclinical, with clinical findings often consisting of heart failure, occurring in 10–15% of patients.^1^ Severe systolic failure does occur in IIM, but diastolic dysfunction is more common, estimated to be present in 12–42% of patients.^1^ Because myocardium is a modified skeletal muscle, it is likely that immunological dysfunction is the cause of myocardial complications in IIM.^1^ In up to 30% of IIM patients, myocarditis has been found on autopsy, with histology demonstrating mononuclear inflammatory infiltrates localized to the endomysium with subsequent damage to cardiac myocytes.^1^ A systematic review on cardiac involvement in IIM by Gupta *et al*.^6^ in 2011 found a similar proportion of IIM patients with myocarditis, 38%. Interestingly, IIM is also associated with higher rates of diabetes, hypertension, and coronary disease.^3,9^ In one Australian database of IIM patients, it was reported that the overall prevalence rates of hypertension and diabetes were 62% and 29%, respectively.^9^ Although the use of corticosteroids could be contributing to this higher prevalence, this study also found that hypertension and ischaemic heart disease were more likely to be present before rather than after a diagnosis of IIM is made, suggesting that the inflammatory nature of these disorders may, in itself, be a causative factor in the metabolic syndrome.^9^

Subclinical arrhythmias and conduction abnormalities are also a frequent finding, occurring in 30–80% of patients.^1^ The abnormalities most commonly found on electrocardiogram are premature ventricular complexes, heart block, and non-specific ST and T wave changes.^6^ Additionally, a recent study demonstrated that patients with dermatomyositis and polymyositis had longer corrected QT intervals compared with controls, which may predispose patients to ventricular arrhythmias.^10^ Lastly, pericardial involvement is a possibility with pericarditis or pericardial effusion but appears to be rare.^1,3,6^

Our patient had a muscle biopsy confirming inflammatory myopathy, with macrophage, lymphocytic, and eosinophilic infiltration of the endomysium, but did not have specific biopsy features or myositis-specific antibodies to point towards a specific IIM. Our case is unique given the fact that our patient was largely asymptomatic from a cardiac perspective and had a relatively mild presentation of IIM overall, but presented with a sudden cardiac arrest. As discussed, cardiac involvement in IIM is often subclinical and primarily presents with diastolic dysfunction.^6^ Cardiac arrest has been reported only in a few other case studies.^1,3,6,7^ In addition, these case studies have often described patients with well-differentiated seropositive IIM.^1,3,6,7^ The patients in these few cases also demonstrated clinically significant cardiac complications before the presentation of cardiac arrest, often of systolic or diastolic heart failure.^1,3,6,7^ In hindsight, perhaps the baseline ECG, showing intraventricular conduction delay, as well as the baseline echocardiogram, which revealed a wall motion abnormality in the basal inferolateral region, was an indication of pre-existing myocarditis from IIM before the presentation of the arrest. Additionally, the cMRI revealed LGE in the same basal inferolateral region of the previous baseline echocardiogram, possibly indicating that the myocardial scar was present before the presentation of the cardiac arrest. However, our patient also presented with atypical symptoms consisting of occasional myalgias and a lack of muscle weakness, making the original diagnosis of IIM challenging. Studies have shown that cardiovascular involvement can occur at any stage of the disease process in IIM, even when other manifestations of IIM are less severe.^1^

**Figure 4:**
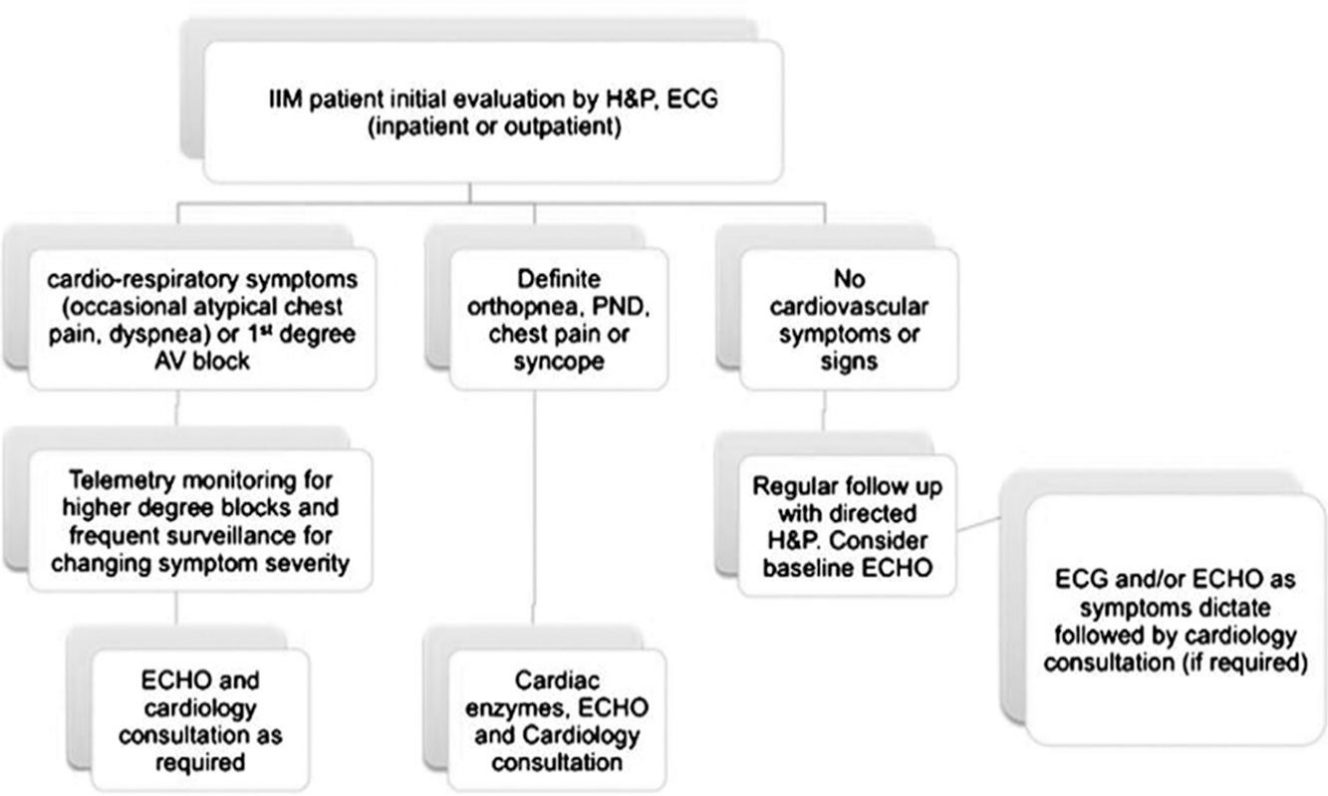
Idiopathic inflammatory myopathy evaluation algorithm. Algorithm adapted from Gupta *et al*.^6^ for determining cardiac involvement in idiopathic inflammatory myopathy.

Currently, there are no guidelines related to the diagnosis or treatment of the cardiovascular manifestations of IIM. A systematic review on the cardiovascular complications of IIM by Gupta et al.^6^ makes a recommendation that any IIM patient should have a thorough cardiac history and physical and baseline electrocardiogram (*Figure 4*). If symptoms are present, they recommend further investigation, such as Holter and echocardiogram.^6^ Additionally, a baseline echocardiogram may be considered for some patients.^6^

Treatment recommendations are similarly based on expert opinion and case series, given the lack of guidelines. A recent study of 15 patients with polymyositis and dermatomyositis with cMRI-confirmed myocarditis demonstrated that glucocorticoid monotherapy resulted in a remission of skeletal inflammation, but did not result in improvement of the myocarditis.^11^ Therefore, corticosteroid monotherapy is probably insufficient for myocarditis associated with IIM, and other clinicians have used combinations of immunosuppressive medication including azathioprine, hydroxychloroquine, cyclophosphamide, and rituximab along with prednisone.^1^ Intravenous immunoglobulin was used in our patient during hospital admission, and has also been used in other patients with severe myositis, with one patient demonstrating complete recovery of myocardial damage and EF.^1,12^

In terms of ICD therapy, although there are no specific guidelines pertaining to the IIM population, European Society of Cardiology (ESC) guidelines do provide guidance with regard to myocarditis, including a Class IIa recommendation for ICD implantation before hospital discharge in patients presenting with VF in the context of acute myocarditis, which was performed in our patient.^4^

There are some diagnostic limitations in the case of our patient that should be acknowledged. Although the clinical presentation and cMRI findings certainly suggest myocarditis secondary to IIM as the most likely aetiology, it must be noted that another cause such as viral myocarditis could not be fully excluded in our patient. cMRI was extremely useful in the diagnosis of our patient by revealing LGE of the basal inferolateral wall, which was predominantly mid-myocardial. Mid-myocardial LGE, along with sub-epicardial LGE, is commonly seen in myocarditis, whereas ischaemic cardiomyopathy most often presents with sub-endocardial LGE with variable degrees of transmurality usually following a coronary distribution.^13,14^ In actuality, endomyocardial biopsy is the gold standard for establishing the diagnosis of myocarditis and the underlying aetiology.^15^ Endomyocardial biopsy was discussed in our patient case, but given the lack of increased T2 signal in the cMRI to indicate active inflammation, the utility of biopsy was thought to be low. It should also be acknowledged that 8–22% of patients presenting with myocarditis may have gene variants implicated in dilated cardiomyopathy and non-dilated left ventricular cardiomyopathy,^5^ and that genetic testing in our patient may have identified such a gene. However, although genetic testing was discussed in our case, it was not conducted, as it was unlikely to change the patient’s treatment plan. Lastly, with regard to targeted temperature management, our patient presented before the latest guidelines were formulated in this area, which now suggest focusing on prevention of hyperthermia rather than inducing hypothermia in patients.^16^

In summary, our case demonstrates that cardiac arrest is a rare but possible complication of IIM, and can be the first cardiac manifestation of the condition. Cardiac complications of IIM may occur more frequently than previously thought, and mainly comprise of cardiomyopathy, myocarditis, conduction abnormalities, and tachyarrhythmias. IIM patients require a thorough evaluation for cardiac symptomatology and a low threshold for additional investigations, such as Holter and echocardiogram. There is a need for collecting more evidence on screening, diagnosis, and treatment of the cardiac complications of IIM.

## Supplementary Material

ytad589_Supplementary_Data

## Data Availability

The data underlying this article are available in the article and in its online Supplementary material.

## Lead author biography

Varun Srivatsav is currently a cardiology resident at Queen’s University in Canada. Varun Srivatsav completed his Internal Medicine residency at the University of Saskatchewan in June 2023. He completed his medical school training at the Michael G. DeGroote School of Medicine, McMaster University, graduating in 2019. His scientific interest is in cardiac imaging and echocardiography.
